# Investigation of the novelty of latent functionally thermal fluids as alternative to nanofluids in natural convective flows

**DOI:** 10.1038/s41598-020-77224-6

**Published:** 2020-11-20

**Authors:** Zoubida Haddad, Farida Iachachene, Eiyad Abu-Nada, Ioan Pop

**Affiliations:** 1 Institute of Electrical and Electronic Engineering, University M’hamed BOUGARA of Boumerdes, Boumerdes, Algeria; 2 Faculté des Hydrocarbures et de la Chimie, Université M’Hamed BOUGARA de Boumerdes, Boumerdes, Algeria; 3 Department of Mechanical Engineering, Khalifa University, Abu Dhabi, United Arab Emirates; 4 Department of Applied Mathematics, Babeş-Bolyai University, 400084 Cluj-Napoca, Romania

**Keywords:** Fluid dynamics, Applied mathematics

## Abstract

This paper presents a detailed comparison between the latent functionally thermal fluids (LFTFs) and nanofluids in terms of heat transfer enhancement. The problem used to carry the comparison is natural convection in a differentially heated cavity where LFTFs and nanofluids are considered the working fluids. The nanofluid mixture consists of Al_2_O_3_ nanoparticles and water, whereas the LFTF mixture consists of a suspension of nanoencapsulated phase change material (NEPCMs) in water. The thermophysical properties of the LFTFs are derived from available experimental data in literature. The NEPCMs consist of n-nonadecane as PCM and poly(styrene-*co*-methacrylic acid) as shell material for the encapsulation. Finite volume method is used to solve the governing equations of the LFTFs and the nanofluid. The computations covered a wide range of Rayleigh number, 10^4^ ≤ Ra ≤ 10^7^, and nanoparticle volume fraction ranging between 0 and 1.69%. It was found that the LFTFs give substantial heat transfer enhancement compared to nanofluids, where the maximum heat transfer enhancement of 13% was observed over nanofluids. Though the thermal conductivity of LFTFs was 15 times smaller than that of the base fluid, a significant enhancement in thermal conductivity was observed. This enhancement was attributed to the high latent heat of fusion of the LFTFs which increased the energy transport within the cavity and accordingly the thermal conductivity of the LFTFs.

## Introduction

Since the energy reserves in the world reduce continuously, there is a growing need to find alternate sources of energy. The heat transfer enhancement in thermal engineering systems is one of these attempts. Heat transfer fluids (HTF) such as engine oil, ethylene glycol and water possess very low thermal conductivity when compared to solids. Therefore, the dispersion of solid nanoparticles with large thermal conductivity is an efficient way to improve thermal properties of such fluids^1^. The thermal conductivity of HTF can be enhanced using this method^2,3^. In this regard, various types of metallic and non-metallic nanoprticles like Cu, Ag, Al_2_O_3_, TiO_2_, CuO, SiO_2_, CNTs have been used in literature, resulting in suspensions called nanofluids^4^. A very important application of HTF is in thermal energy storage where the latent heat of melting can be used to store considerable amount of energy in materials known as phase change materials (PCMs) and then this stored energy can be released later in cases of high energy demands. This is widely used technology in solar energy, where the energy is normally stored during the day hours and released back to the solar assisted power plants at night^5^.

Several studies reported the effect of adding nanoparticles, without liquid–solid phase change in the core, on the enhancement of convective heat transfer flows. Examples can be found in^6–22^. Other work studies on nanofluids can be also found in^23–30^. In particular, for natural convection applications, some results have shown the effectiveness of dispersing nanoparticles in a base fluid^31,32^, while some other results showed a negative effects^33,34^. This indicates that the role of nanoparticles in natural convective flows is still a controversy^35^. Nowadays, the advances in nanotechnology made the encapsulation of PCMs at nanoscale possible. Actually, the PCM capsule size plays a substantial role and could push the research frontiers of PCM encapsulation applications in heat exchangers, thermal storage systems, and thermal control systems^36^. Nanoencapsulation is a combination of different processes in which droplets or particles are coated with a continuous layer to produce capsules in a nanometer size, named nanocapsule. Particularly, nanoencapsulated PCMs (NEPCMs) are made of two principle parts, which are: a PCM as active or core material and an organic or polymer shell as a wall material^37,38^. Several types of organic or inorganic solid–liquid NEPCMs were reported in the literature. Choi et al.^39^ prepared a NEPCM by using amelamine formaldehyde shell and tetradecane as PCM. Fang et al.^40^ used a urea formaldehyde shell to encapsulate tetradecan PCM. Yang et al.^41^ employed in situ polymerization to fabricate poly(methyl methacrylate), poly(ethyl methacrylate) polystyrene and (ethyl methacrylate) capsules containing tetradecane. Wu et al.^42^ prepared a NEPCM by using polymer shell and paraffin as PCM. Alay et al.^43^ used poly (methylmethacrylate-*co*-glycidyl methacrylate) shell to encapsulate n-hexadecane. Karaipekli et al.^44^ formulated the NEPCMs, using N-nonadecane as PCM and poly (styrene-*co*-methacrylic acid) as shell material for the encapsulation. A detailed review on the nanoencapsulation of inorganic and organic PCMs is presented in the literature^45^.

On the basis of PCMs which possess a significant amount of latent thermal energy capacity near their fusion point, a new technique was recently raised to improve the heat capacity of HTF at the desired temperature range^46^. This technique consists of a combination of well selected NEPCMs such paraffin and HTF like water, resulting in latent functionally thermal fluids (LFTFs). These fluids are mainly divided into two different categories, which are: (1) PCMs nanoemulsions or miniemuslion, where an emulsifier is used to disperse PCMs in water and (2) nanoencapsulated PCM slurries, in which PCMs are nanoencapsulated and then dispersed in water. In the latter, the mixture show high stability and low viscosity^46^. This unique feature of low viscous suspension gives such NEPCMs unique properties in enhancing the heat transfer and concurrently does not cause any adverse effect on pressure drop due to the high level of viscosity encountered in classical nanofluids.

Based on the literature, a huge interest was evinced in the study of natural convection in nanofluids. However, the study of natural convection in LFTFs remains relatively unexplored. The pioneer work of Ghalambaz et al.^47^ introduced a numerical study on natural convection of NEPCMs in a square cavity, using n-octadecane as active material and polymethylmethacrylate (PMMA) as wall material. Several theoretical correlations for thermophysical properties of nanofluids and LFTFs were used. The heat transfer enhancement was found to be mainly dependent on the melting temperature. An enhancement of 10% was observed for non dimensional melting temperature of 0.25. Similar result was found in another study conducted by Hajjar et al.^48^. However, in their study, the temperature at the hot wall is considered time-periodic. Mehryan et al.^49^ presented a numerical study on the natural convective flow of NEPCMs suspensions in an eccentric annulus. Their results showed that increasing the volume fraction of NEPCMs leads to enhance the heat transfer in the annulus. 5% of NEPCM particles is considered as an optimal volume fraction for heat transfer enhancement in the annulus. Zadeh et al.^50^ numerically analyzed the natural convection and entropy generation of NEPCMs, filled in a square enclosure with solid triangular block. It is observed that the Nusselt number and total entropy generation increase with the increment of the NEPCM volume fraction. Zadeh et al.^51^ analyzed the convective heat transfer and entropy generation of NEPCM suspension in a porous square cavity. Their results showed that the heat transfer rate is maximum and the generated entropy is minimum when the fusion temperature of the nano-capsules is equal to 0.5. Besides, adding the nanosized particles of encapsulated phase change materials to the host fluid increases the heat transfer rate up to 45%. Ghalambaz et al.^52^ examined the free convection heat transfer of a NEPCM suspension in an inclined porous cavity. They found that the presence of NEPCMs lead to heat transfer enhancement. The best heat transfer performance occurs for the non-dimensional fusion temperature of 0.5 and inclination angle of 42°. It is also found that a decrease in the Stefan number improves heat transfer. Ghalambaz et al.^53^ numerically studied the thermal and hydrodynamic characteristics of a NEPCM in an annulus of a porous eccentric horizontal cylinder. It is shown that for low Rayleigh number values, the heat transfer rate is maximum when the fusion temperature of the capsule is about 0.5. However, for high Rayleigh numbers, the highest rate of heat transfer can be achieved when the fusion temperature varies between 0.25 and 0.65.

It is clear that the number of studies on natural convection in LFTFs is very limited and to the best of the author’s knowledge, there is no study in literature that focus on the comparison between the heat transfer enhancement using LFTFS and Nanofluids. Accordingly, the present paper takes natural convection in a differentially heated square cavity as a test case. To consider the sensitivity of the results to thermophysical properties variation within the flow field, the thermophysical properties of LFTFs are considered a dual function of nanoparticle volume fraction and temperature. Also, to have the results mimic real life applications, thermophysical properties values are derived from real experimental data rather than using theoretical formulas. This includes thermal conductivity, viscosity, density, and specific heat data. Flow and temperature fields and heat transfer enhancement for both nanofluids and LFTFs as a function of different controlling parameters are investigated numerically in details. The simulations were conducted for Rayleigh numbers varying from 10^4^ to 10^7^ and nanoparticle volume fraction ranging between 0 and 1.69%.

## Mathematical model

### Physical problem

**Figure 1:**
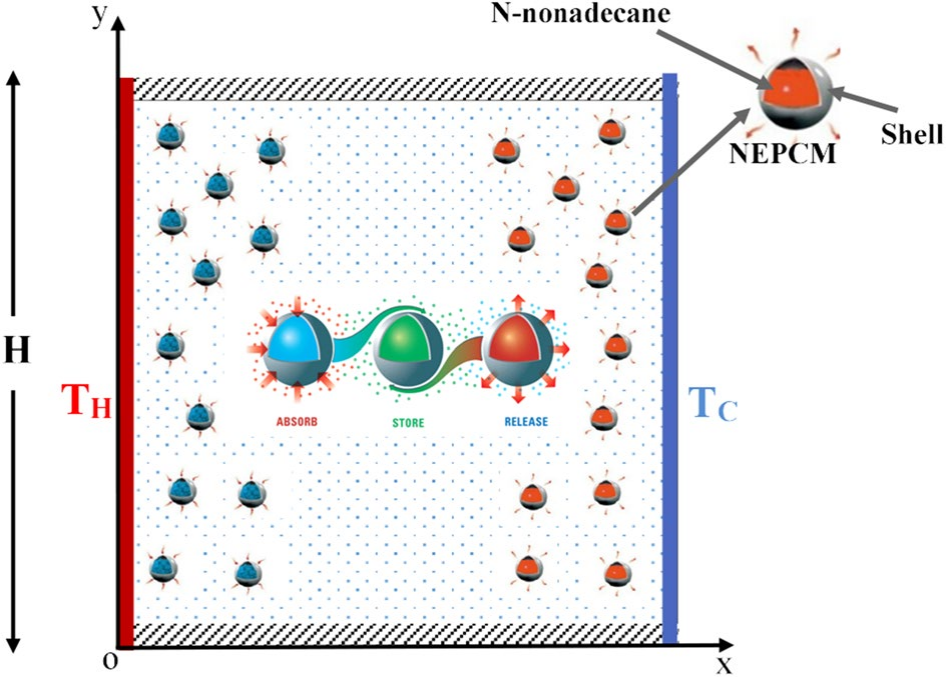
Schematic of the physical model.

A square enclosure, of length H, filled with a mixture of water and dispersed NEPCMs or Al_2_O_3_ nanaoparticles is shown in Fig. 1. The NEPCMs are made of n-nonadecane as a core and styrene/methacrylic acid copolymer as shell. The PCM undergoes a solid–liquid phase change at the melting temperature. A schematic of the physical model with coordinate system is presented in Fig. 1. As shown, the vertical walls are maintained at hot and cold isothermal temperatures, while the horizontal walls are kept adiabatic. Based on the experimental study of Karaipekli et al.^44^, such LFTFs are considered stable without any stabilizing agent or emulsifying and well dispersed with zeta potential of − 56.8 mV.

To model natural convection of LFTFs or nanofluids some assumptions are used in the current study. The mixture is assumed to be in thermal equilibrium, the flow is incompressible, Newtonian, and steady. The vibration of whole mixture density in the flow field is assumed to follow the Boussinesq approximation.

Following the above assumptions, the governing equations for the problem, which are continuity, momentum, and energy equations can be written respectively as

1 $$\frac{{\partial\left( \uprho \text{u} \right)}}{{\partial\text{x}}} + \frac{{\partial \left( \uprho \text{v} \right)}}{{\partial\text{y}}} = 0$$

2 $$\frac{\partial}{{\partial\text{x}}}\left( {{\uprho \text{uu}}} \right) + \frac{\partial}{{\partial\text{y}}}\left( {{\uprho \text{uv}}} \right) = - \frac{{\partial\text{p}}}{{\partial\text{x}}} + \frac{\partial}{{\partial\text{x}}}\left( {\upmu \frac{{\partial\text{u}}}{{\partial\text{x}}}} \right) + \frac{\partial}{{\partial\text{y}}}\left( {\upmu \frac{{\partial\text{u}}}{{\partial\text{y}}}} \right)$$

3 $$\frac{\partial}{{\partial\text{x}}}\left( {{\uprho \text{uv}}} \right) + \frac{\partial}{{\partial\text{y}}}\left( {{\uprho \text{vv}}} \right) = - \frac{{\partial\text{p}}}{{\partial\text{y}}} + \frac{\partial}{{\partial\text{x}}}\left( {\upmu \frac{{\partial\text{v}}}{{\partial\text{x}}}} \right) + \frac{\partial}{{\partial\text{y}}}\left( {\upmu \frac{{\partial\text{v}}}{{\partial\text{y}}}} \right) + \uprho \upbeta \text{g}(\text{T} - \text{T}_{\text{C}} )$$

4 $$\frac{\partial}{{\partial\text{x}}}\left( {{\uprho \text{uT}}} \right) + \frac{\partial}{{\partial\text{y}}}\left( {{\uprho \text{vT}}} \right)= \frac{\partial}{{\partial\text{x}}}\left( {\frac{{\rm k}}{{{\rm C}_{{\rm p}} }}\frac{{\partial\text{T}}}{{\partial\text{x}}}} \right) + \frac{\partial}{{\partial\text{y}}}\left( {\frac{{\rm k}}{{{\rm C}_{{\rm p}} }}\frac{{\partial\text{T}}}{{\partial\text{y}}}} \right)$$

Note that thermal conductivity, viscosity, density, and specific heat are considered to vary in the flow field as shown in the governing equations. Also, note that the above equations are valid for both nanofluids and LFTFs.

### Thermophysical properties of the LFTF

**Figure 2:**
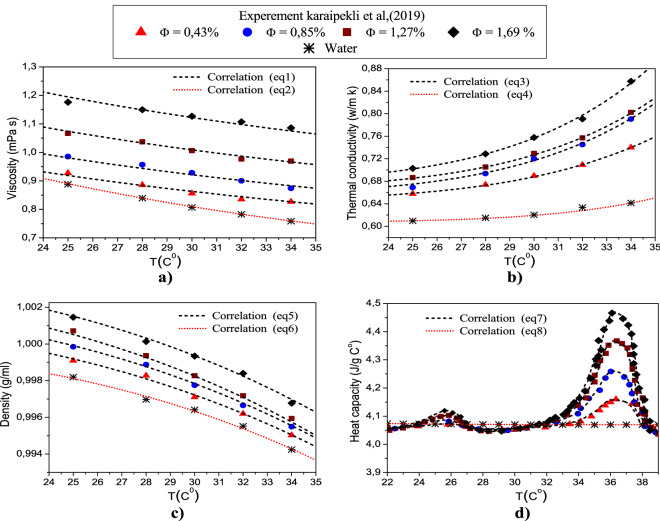
Thermophysical proprieties of the LFTFs.

The correlations for the specific heat capacity, dynamic viscosity, density and thermal of the LFTF and water are derived using the available experimental data of Karaipekli et al.^44^. The correlations (Eqs. 5–12) are derived in the present work, using curve fitting technique by the method of least squares. The R^2^ value is 99.8% and a maximum error is 1%. As seen in Fig. 2, the experimental data are in good accordance with the estimated values from the correlations. In Karaipekli et al.^44^ study, the NEPCMs were prepared using N-nonadecane as PCM and poly (styrene-*co*-methacrylic acid) as shell material for the encapsulation. The average size of the NEPCMs is equal to 212 nm, which is in the typical range of nanosized phase change particles (1–1000 nm)^44^. The LFTFs were prepared at different volume fractions of 0.43, 0.85, 1.27 and 1.69%, and hence it can be considered as a dilute suspension. The density, thermal conductivity and dynamic viscosity correlations are valid in the temperature range of 25–34 °C, while the specific heat capacity correlation is valid in the range of 25–39 °C.

The correlations we derived in the current study for the dynamic viscosity, density, specific heat capacity, and thermal conductivity of both LFTF and water are expressed, respectively, as follows:

*Dynamic viscosity*

5 $$\upmu _{{{\rm LFTF}}} = (2{.838 + 0}{.3753}{\upphi}^{{{1}{.79}}} {)}\,{\rm (1 + T)}^{{{\rm - 0}{\rm .355}}}$$

6 $$\upmu _{{\rm w}} = 1{\rm .5826 - 3}{\rm .74 \times 10}^{{{\rm - 2}}} {\rm T + 3}{\rm .879 \times 10}^{{{\rm - 4}}} {\rm T}^{{\rm 2}}$$

*Thermal conductivity*

7 $${\rm k}_{{{\rm LFTF}}} = (0{\rm .63456 + 2}{\rm .5 \times 10}^{{{\rm - 2}}} {\upphi) + ( - 1}{\rm .39 + 10}{\rm .99}\upphi - 10{\rm .59}\upphi^{{\rm 2}} {\rm + 3}{\rm .3466}\upphi^{{\rm 3}} {\rm )10}^{{{\rm - 11}}} {\rm T}^{{{\rm 6}{\rm .37}}}$$

8 $${\rm k}_{{\rm w}} = 0{\rm .607 + 1}{\rm .26 \times 10}^{{{\rm - 14}}} {\rm T}^{{{\rm 8}{\rm .12}}}$$

*Density*

9 $$\uprho _{{{\rm LFTF}}} = (1{\rm .0008 + 2}{\rm .03 \times 10}^{{{\rm - 3}}} {\upphi) + ( - 10}{\rm .79 + 8}{\rm .84}\upphi - 11{\rm .536}\upphi^{{\rm 2}} {\rm + 4}{\rm .063}\upphi^{{\rm 3}} {\rm )10}^{{{\rm - 8}}} {\rm T}^{{{\rm 3}{\rm .18}}}$$

10 $$\uprho _{{\rm w}} = {1}{\rm .00017 - 3}{\rm .29 \times 10}^{{{\rm - 8}}} {\rm T}^{{{\rm 3}{\rm .43}}}$$

*Specific heat capacity*

11 $${\rm (c}_{{\rm p}} {\rm )}_{{{\rm LFTF}}} = {\rm c}_{{\rm 0}}+ {\rm Ae}^{{{\rm B(T - T}_{{\rm c}} {\rm )}^{{\rm 2}} }}$$

For $${\text{T}} < 30\,^{{\circ }} {\text{C}}$$

$$\left\{ {\begin{array}{*{20}l} {{\rm c}_{{\rm o}} = {\rm 4}{\rm .047}} \hfill \\ {{\rm A = 3}{\rm .86}\,{\rm \times 10}^{{{\rm - 3}}} {\rm + 3}{\rm .944 \times 10}^{{{\rm - 2}}} \,{\upphi}} \hfill \\ {{\rm B = 9}{\rm .698}\,{\rm \times 10}^{{{\rm - 2}}} {\rm - 0}{\rm .56568}\,\upphi+ 0{\rm .16672}\,{\upphi}^{{\rm 2}} } \hfill \\ {{\rm T}_{{\rm c}} = {\rm 25}{\rm .35}} \hfill \\ \end{array} } \right.$$

For $${\text{T}} \ge 30\,^{{\rm \circ }} {\text{C}}$$

$$\left\{ {\begin{array}{*{20}l} {{\rm c}_{{\rm o}} = {\rm 4}{\rm .054}} \hfill \\ {{\rm A = - 6}{\rm .667 \times 10}^{{{\rm - 3}}} {\rm + 0}{\rm .2555}\,{\upphi}} \hfill \\ {{\rm B }= - 0{\rm .25721 - 0}{ .2252\upphi + 9}{\rm .708}\,{\rm \times 10}^{{{\rm - 2}}} {\upphi}^{{\rm 2}} } \hfill \\ {{\rm T}_{{\rm c}} = {\rm 36}{\rm .24}} \hfill \\ \end{array} } \right.$$

12 $${\rm (c}_{{\rm P}} {\rm )}_{{\rm w}} ={\rm 4}{\rm .09095 - 8}{\rm .37 \times 10}^{{{\rm - 4}}} \,{\rm T - 2}{\rm .9}\,{\rm \times 10}^{{{\rm - 6}}} \,{\rm T}^{{\rm 2}} + \,{\rm 2}{\rm .67}\,{\times 10}^{{{\rm - 7}}} \,{\rm T}^{{\rm 3}}$$

where the subscript w stand for water as a base fluid. Note that the temperature is °C, while the nanoparticle volume fraction is in percentage (%).

Equations (1)–(4) can be expressed in a non-dimensional form using the following dimensionless parameters:

13 $$\begin{aligned} {\rm X} & = \frac{{\rm x}}{{\rm H}}{\rm ,Y = }\frac{{\rm y}}{{\rm H}}{\rm ,U = }\frac{{{\rm uH}}}{{\upalpha _{{{\rm w,ref}}} }}{\rm ,V = }\frac{{{\rm vH}}}{{\upalpha _{{{\rm w,ref}}} }}{\rm ,P = }\frac{{{\rm pH}^{{\rm 2}} }}{{\uprho _{{{\rm w,ref}}} \upalpha _{{{\rm w,ref}}}^{{\rm 2}} }}{\rm ,\uptheta = }\frac{{{\rm T - T}_{{\rm C}} }}{{{\rm T}_{{\rm H}} {\rm - T}_{{\rm C}} }}{\rm ,\uprho }^{{\rm *}} = \frac{{\uprho _{{{\rm LFTF}}} }}{{\uprho _{{{\rm w,ref}}} }}{\rm ,} \\ {\rm c}_{{\rm p}}^{{\rm *}} & = \frac{{{\rm (c}_{{\rm p}} {\rm )}_{{{\rm LFTF}}} }}{{{\rm (c}_{{\rm p}} {\rm )}_{{{\rm w,ref}}} }}{\rm ,k}^{{\rm *}} = \frac{{{\rm k}_{{{\rm LFTF}}} }}{{{\rm k}_{{{\rm w,ref}}} }}{,\upmu }^{{\rm *}} = \frac{{\upmu _{{{\rm LFTF}}} }}{{\upmu _{{{\rm w,ref}}} }} \\ \end{aligned}$$

where the subscript “ref” indicates the reference temperature which is equal to T_C_.

By using the non-dimensional parameters, the continuity, momentum and energy equations are written as

14 $$\frac{{\partial\left( {\uprho ^{{\rm *}} \,{\rm U}} \right)}}{{\partial\text{X}}} + \frac{{\partial\left( {\uprho ^{{\rm *}} \,{\rm V}} \right)}}{{\partial\text{Y}}}={\rm 0}$$

15 $$\frac{\partial}{{\partial\text{X}}}\left( {\uprho ^{{\rm *}} {\rm UU}} \right) + \frac{\partial}{{\partial\text{Y}}}\left( {\uprho ^{{\rm *}} \,{\rm UV}} \right)= - \frac{{\partial\text{P}}}{{\partial\text{X}}}{\rm + Pr}\left[ {\frac{\partial}{{\partial\text{X}}}\left( {\upmu ^{{\rm *}} \,\frac{{\partial\text{U}}}{{\partial\text{X}}}} \right) + \frac{\partial}{{\partial\text{Y}}}\left( {\upmu ^{{\rm *}} \,\frac{{\partial\text{U}}}{{\partial\text{Y}}}} \right)} \right]$$

16 $$\frac{\partial}{{\partial\text{X}}}\left( {\uprho ^{{\rm *}} \,{\rm UV}} \right) + \frac{\partial}{{\partial\text{Y}}}\left( {\uprho ^{{\rm *}} \,{\rm VV}} \right)= - \frac{{\partial\text{P}}}{{\partial\text{Y}}}{\rm + Pr}\left[ {\frac{\partial}{{\partial\text{X}}}\left( {\upmu ^{{\rm *}} \frac{{\partial\text{V}}}{{\partial\text{X}}}} \right) + \frac{\partial}{{\partial\text{Y}}}\left( {\upmu ^{{\rm *}} \,\frac{{\partial\text{V}}}{{\partial\text{Y}}}} \right)} \right]{\rm + RaPr\uptheta (\uprho }^{{\rm *}} \,\upbeta ^{{\rm *}} {\rm )}$$

17 $$\frac{\partial}{{\partial\text{X}}}\left( {\uprho ^{{\rm *}} \,{\rm U\uptheta }} \right) + \frac{\partial}{{\partial\text{Y}}}\left( {\uprho ^{{\rm *}} \,{\rm V\uptheta }} \right)= \frac{\partial}{{\partial\text{X}}}\left( {\frac{{{\rm k}^{{\rm *}} }}{{{\rm c}_{{\rm p}}^{{\rm *}} }}\frac{{\partial{ \uptheta }}}{{\partial\text{X}}}} \right) + \frac{\partial}{{\partial\text{Y}}}\left( {\frac{{{\rm k}^{{\rm *}} }}{{{\rm c}_{{\rm p}}^{{\rm *}} }}\frac{{\partial{ \uptheta }}}{{\partial\text{Y}}}} \right)$$

where ${\rm Ra = }\frac{{{\rm g}\upbeta _{{\rm w}} {\rm (T}_{{\rm h}} {\rm - T}_{{\rm c}} {\rm )L}^{{\rm 3}} }}{{\upnu _{{{\rm w,ref}}} \upalpha _{{{\rm w,ref}}} }}$ and ${\rm Pr = }\frac{{\upnu _{{{\rm w,ref}}} }}{{\upalpha _{{{\rm w,ref}}} }}$ are the Rayleigh and Prandtl numbers, respectively.

The local and mean Nuselt numbers along the hot wall are expressed, respectively, as follows:

18 $${\rm Nu = - }\frac{{{\rm k}_{{{\rm LFTF}}} }}{{{\rm k}_{{\rm w}} }}\left. {\frac{{\partial{ \uptheta }}}{{\partial\text{X}}}} \right|_{{{\rm x = 0}}}$$

19 $${\rm Nu}_{{{\rm avr}}} = \int\limits_{{\rm 0}}^{{\rm 1}} {{\rm Nu}\,{\rm dY}} {\rm .}$$

## Numerical method

The governing Eqs. (14)–(17) with the respective boundary conditions are solved using the finite volume method. The diffusion terms in the governing equations (in momentum and energy) are approximated using a second order accurate central differencing scheme whereas a second-order up-wind difference scheme is used to discretize the convective terms. The SIMPLE algorithm is used to solve the pressure–velocity coupling. To obtain converged solution, an under-relaxation scheme has been employed. The convergence criterion was set such that the absolute residual is restricted to be smaller than 10^–9^.

## Validation

**Figure 3:**
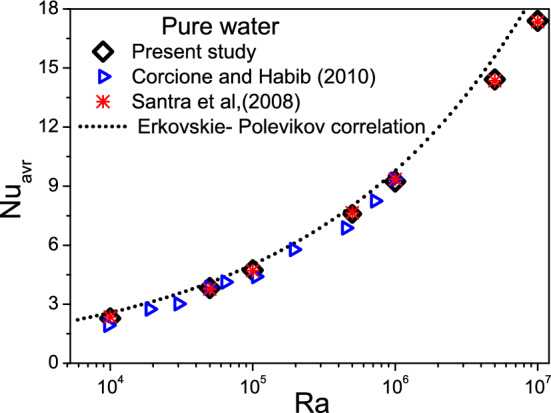
Average Nusselt number variation with Rayleigh number.

**Figure 4:**
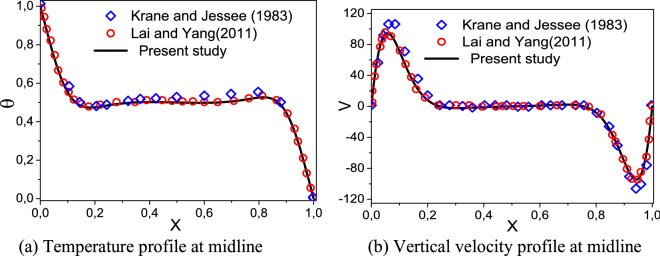
Comparison between present work with other published data (Ra = 1.89 × 10^5^ and Pr = 0.7).

In order to test the reliability of the mathematical model as well as the numerical method, several comparisons have been made for the case of natural convection in a differentially heated square cavity filled with pure fluid. Figure 3 depicts the mean Nusselt number versus Rayleigh number obtained from the Erkovskie- Polevikov correlations^54,55^, numerical data by Santra et al.^56^ and Corcione and Habib^57^. The present numerical solution is also validated against the Krane and Jessee experiment^58^ and numerical results of Yang and Lai^59^ as shown in Fig. 4a,b. It is clear from these figures that the present code is in good agreement with the reported data.

**Figure 5:**
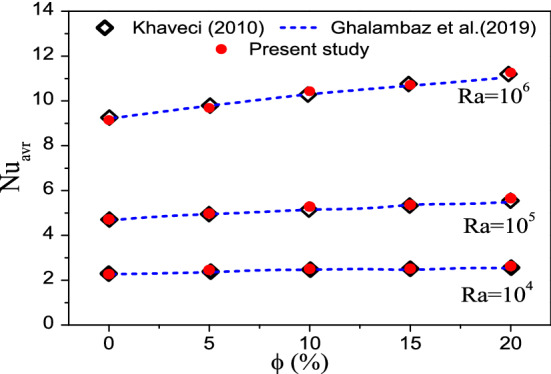
Comparison between the average Nusselt number of the present study and those reported in the literature.

Another comparison for the case of natural convective flow in a square cavity filled with water–Al_2_O_3_ nanofluids^47,60^ is presented in Fig. 5. The comparison of the computed average Nusselt numbers with those reported in the literature for different Rayleigh numbers and naoparticle volume fractions clearly shows a good concordance.

### Grid independency test

A grid independence test is used to determine the appropriate grid size for the numerical simulations. The cavity is meshed with a non-uniform rectangular grid with a very fine spacing near the walls. Grid sizes of 60 × 60, 80 × 80, 100 × 100, 120 × 120, 140 × 140, and 160 × 160 are used to simulate the flow within the cavity, filled with water at Ra = 10^5^. Table 1 depicts the mean Nusselt number for various grid points. As it can be seen, a 120 × 120 can ensure a grid independent solution with a deviation of 0.7%. Therefore, this grid is employed in the present study.

**Table 1:** The grid size effect on the average Nusselt number for Ra = 10^5^.

Grid size	60 × 60	80 × 80	100 × 100	120 × 120	140 × 140	160 × 160
Nuavr	5.381	5.380	5.379	5.378	5.378	5.378

## Results and discussion

### Thermophysical properties of LFTFs and nanofluids

In this part, the thermophysical properties of both LFTFs and nanofluids are compared and discussed for particle volume concentration between 0.43 and 1.69%, and in the temperature range from 22 to 39 °C. The thermophysical properties of the LFTFs are reported by Karaipekli et al.^44^, while the thermophysical properties of the nanofluids are calculated based on different formulas commonly adopted in the literature. The nanofluid is considered as mixture of pure water and Al_2_O_3_ nanoparticles. The choice of this type of nanoparticle for the current study is based on the large set of data presented in the literature. Note that the thermal conductivity, specific heat, and density of Al_2_O_3_ nanoparticle are equal to 40 W/mK, 765 J/kg K, and 3970 kg/m^3^, respectively^53^.

- Density

**Figure 6:**
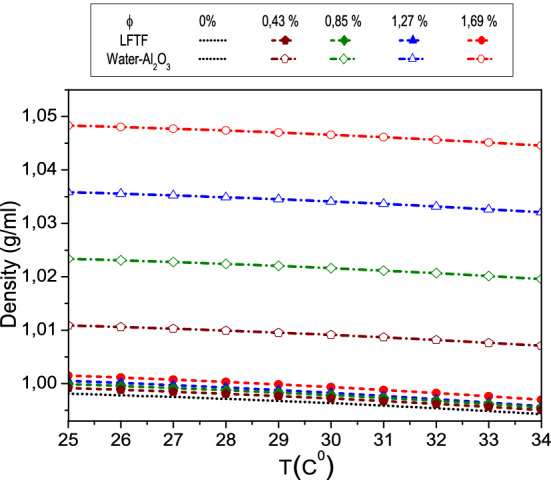
Comparison between The density of LFTFs and nanofluids.

Figure 6 presents the measured density of the LFTFs as function of the temperature and particle volume fraction^44^, and also the density computed on the basis of the mixture theory, which is written as^61^

20 $$\uprho _{{{\rm nf}}} = \left( {{\rm 1} - {\upphi}} \right)\uprho _{{\rm f}} + \upphi{\uprho }_{{\rm s}}$$

As expected, at temperature range between 25 and 34 °C, the density of the nanofluid and LFTFs decreases slightly with increasing temperature. The density decrease rate is similar for both mixtures and it does not exceed 0.4%. This indicates that both densities are approximately independent of temperature. Moreover, it can be seen that both densities increase with increasing particle volume fraction. This increase is insignificant for the LFTFs. For example, at $\upphi = 1.69\%$, the increase in density for the nanofluids and LFTFs is 5% and 0.4%, respectively. This result indicates that the LFTFs can be considered as an iso-density mixture ($\uprho _{{{\rm NEPCM}}} {\rm \approx \uprho }_{{\rm f}}$).

It should be noted that one of the effective way to decrease the sedimentation of nanoparticles in the conventional fluid, and hence improve the stability of the mixture is to lessen the density difference between the base fluid and nanoparticles, which is the case of the present LFTFs and therefore this is considered as a unique feature of the LFTFs.

- Specific heat capacity

**Figure 7:**
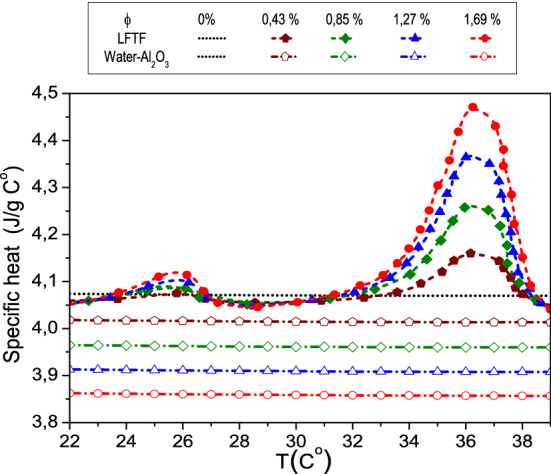
Comparison between the specific heat capacity of LFTFs and nanofluids.

Figure 7 depicts the specific heat capacity of the LFTFs, water and nanofluids. The specific heat capacity of the LFTFs is computed from the following equation, where the values of are determined experimentally^44^:

21 $$\left( {{\rm C}_{{\rm p}} } \right)_{{{\rm LFTF}}} = \frac{{\left( { 1 - \upphi} \right)\uprho _{{\rm f}} \left( {{\rm C}_{{\rm p}} } \right)_{{\rm f}} + \upphi{\uprho }_{{{\rm NEPCM}}} \left( {{\rm C}_{{\rm p}} } \right)_{{{\rm NEPCM}}} }}{{\left( {1 - {\upphi }} \right)\uprho _{{\rm f}} + {\upphi}\uprho _{{{\rm NEPCM}}} }}$$

The specific heat capacity of the nanofluid is expressed as^61^:

22 $$\left( {{\rm C}_{{\rm p}} } \right)_{{{\rm nf}}} = \frac{{\left( { 1 - {\upphi}} \right)\uprho _{{\rm f}} \left( {{\rm C}_{{\rm p}} } \right)_{{\rm f}} + {\upphi}\uprho }_{{\rm s}} \left( {{\rm C}_{{\rm p}} } \right)_{{\rm s}} }{{{\left( { 1 - {\upphi}} \right)\uprho _{{\rm f}} + {\upphi}\uprho }_{{\rm s}} }}$$

It is evident from the figure that the specific heat capacity values of the nanofluids decrease linearly with nanoparticle volume fraction. These values are independent of temperature. However, regarding the LFTFs, the specific heat capacity values are mainly affected by the temperature. In the temperature range between 22 and 28 °C, the specific heat capacities increases slightly since the PCM experiences solid–solid phase change. However, a sharp increase is observed between 28 and 39 °C, where the solid–liquid phase change occurred. This means that the phase change transition plays a critical role on the determination of the heat capacity of the LFTFs. Moreover, it is seen that contrary to nanofluids, increasing volume fraction leads to an improvement in the specific heat capacity of the LFTFs. The heat capacities of the nanofluids (LFTFs) are lower (higher) relative to the base fluid. For instance, at $\upphi = 1.69\%$ and T = 36 °C, the maximum specific heat increase is 10%, while the specific heat of the nanofluid is reduced by 4%, compared to the base fluid. Note that the maximum deviation between the specific heat of the LFTFs and nanofluids is almost 16%.

- Dynamic viscosity

**Figure 8:**
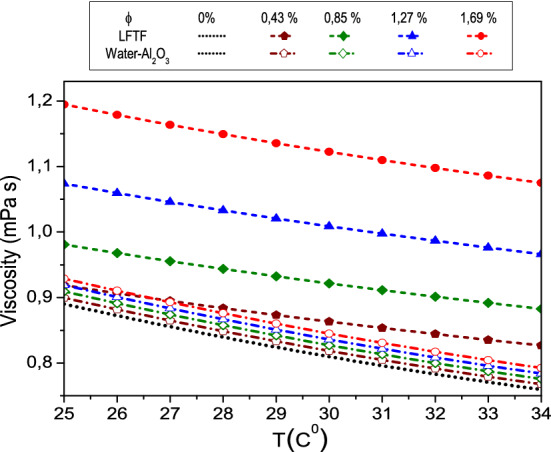
Comparison between the dynamic viscosity of LFTFs and nanofluids.

Figure 8 presents the measured data of the dynamic viscosity of LFTFs^44^. Also, included are the results calculated on the basis of Brinkmann model^61^, which is expressed as following:

23 $$\mu_{nf} = \frac{{\mu_{f} }}{{\left( {1 - \phi } \right)^{2.5} }}$$

As expected, the dynamic viscosity of both mixtures decreases as the temperature increases. However, increasing the volume fraction of the NEPCMs and Al_2_O_3_ nanoparticles leads to an increase in the dynamic viscosity. This increase is more pronounced for LFTFs. For instance, at and T = 34 °C, and compared to water, the maximum increase in the dynamic viscosity of the LFTFs and nanofluids is about 40% and 4%, respectively. This means that the LFTFs show a significant unfavorable increase in viscosity compared to nanofluids.

It should be noted that the benefit of nanofluids as regards to of heat transfer enhancement is related to the compromise between viscosity increase and thermal conductivity increase, therefore, it maybe questionable whether the viscosity enhancement when using LFTFs can be considered as a drawback. This point will be further discussed next when we present the heat transfer characteristics.

- Thermal conductivity

**Figure 9:**
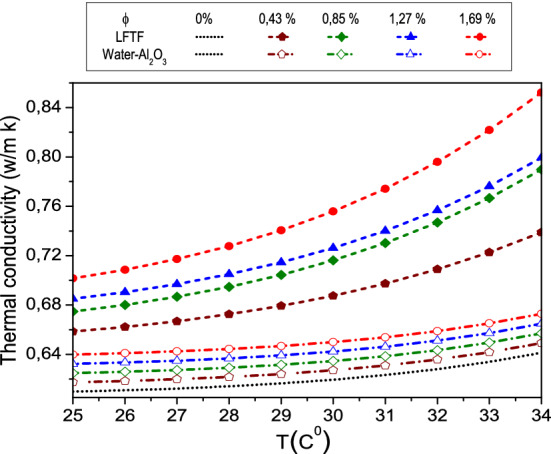
Comparison between the thermal conductivity of LFTFs and nanofluids.

Finally, Fig. 9 shows the measured thermal conductivity of the LFTFs along with the data from Maxwell model, which can be expressed as^61^:

24 $${\rm k}_{{{\rm nf}}} {/}{\rm k}_{{\rm f}} = \frac{{{\rm k}_{{\rm s}} {\rm + 2k}_{{\rm f}} {\rm + 2}\upphi\left( {{\rm k}_{{\rm s}} {\rm - k}_{{\rm f}} } \right)}}{{{\rm k}_{{\rm s}} {\rm + 2k}_{{\rm f}} - \upphi\left( {{\rm k}_{{\rm s}} {\rm - k}_{{\rm f}} } \right)}}$$

As shown, the thermal conductivities of both LFTFs and nanofluids increase with an increase in both particle volume fraction and temperature. It is observed that LFTFs give higher enhancements in the thermal conductivity than those of nanofluids. For example, at and T = 34 °C, and compared to the base fluid, the thermal conductivity of the LFTFs and nanofluids are improved by 5% and 36%, respectively. This result is consistent with some experimental data found in the literature. Fang et al.^62^ measured the thermal conductivity of LFTFs containing polystyrene encapsulated n-tetradecane nanoparticles. They observed 16% thermal conductivity enhancement at mass concentration of 15% and T = 25 °C. Cingarapu et al.^63^ observed that adding silica encapsulated tin nanoparticles to the base fluid increases the thermal conductivity by 13% at nanoparticle volume fraction of 5%. A maximum thermal conductivity enhancement of 20% was reported by Barlak et al.^64^ when they dispersed polyurethane encapsulated n-nonadecane nanoparticles in different base fluids. According to Karaipekli et al.^44^, the thermal conductivity improvement in LFTFs is mainly attributed to the Brownian motion of the NEPCMs and the interaction between the base fluid and surface functionalized NEPCMs.

Note that a variety of mechanisms for enhanced thermal conductivity of nanofluids have been reported in the literature, including the Brownian motion, liquid layering, clustering, thermophoresis, …etc^65^. For example, Keblinski et al.^66^ supposed that heat is conducted along clusters formed by high thermal conductivity nanoparticles. Yu and Choi^67^ found that a layer formed near solid nanoparticle, and which acts like a bridge between liquid and solid should have higher thermal conductivity. It should be noted that in the present study, the thermal conductivity of the NEPCMs is equal to 0.039 W/m K, which is much less than that of the base fluid. However, an enhancement in thermal conductivity is observed. This indicates that nanolayer and clustering effects cannot be responsible for such thermal conductivity enhancement in LFTFs.

In fact, LFTFs can give new perspectives about the mechanisms behind a thermal conductivity increase in nanofluids, by measuring the thermal conductivity of two different nanofluids, where:

- the nanoparticles and base fluid have the same thermal conductivity (iso-conductivity)
- the thermal conductivity of nanoparticles is less than that of the base fluid.

From our perspective, we argue that all the mechanisms presented above are of minor effect and we believe that the high value of latent heat of fusion of the NEPCMs is the main mechanism for the high energy transport within the cavity. At the hot surface of the wall cavity, the NEPCMs melt and accordingly this phase change phenomenon resulted in a high energy transport from the hot wall of the cavity and the NEPCMs. Thus, the NEPCMs carry such high energy content and release it later in regions in the cavity experiencing low temperature. At such low temperature places a solidification of the NEPCMs occurs and therefore a high energy release will be taking place. This high energy transport enhances substantially the thermal conductivity of the LFTFs and accordingly the heat transfer in the cavity. Our statement is consistent with the experimental findings of Karaipekli et al.^44^, where a suspension of low thermal conductivity of NEPCMs (0.039 W/m K) in water resulted in a thermal conductivity greater than that of the suspension of high conductive Al_2_O_3_nanopartices (k = 40 W/m K) in water.

- Analysis on the validity of the Brinkman and Maxwell models

**Figure 10:**
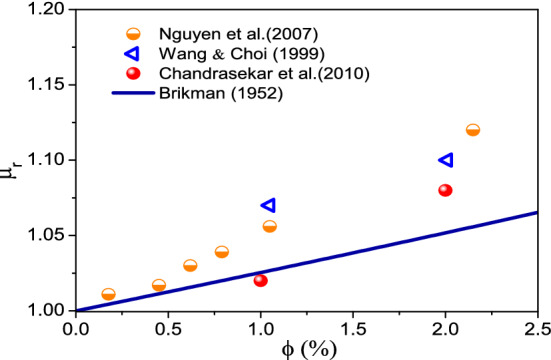
Comparison of the relative viscosity by Brinkman^61^ at different naoparticle concentrations with previous experimental data.

The volume fraction we are using in this study is small (less than 2%). Therefore, the error of using Brinkamn for such values is acceptable. To give more insight on this statement, Fig. 10 depicts the dynamic viscosity ratio of Al_2_O_3_–water nanofluid to the base fluid versus nanoparticle volume fraction up to 2.5%. The experimental data from the previous studies^68–70^ and the data calculated based on the Brinkman model^61^ are presented. It is seen that there exists small discrepancy between the experimental data and the results calculated by Brinkman model^61^. For example, at nanoparticle volume fraction of 2.15%, almost 12% of the viscosity increase is reported by Nguyen et al.^68^, while 5% by the Brinkman model^61^. This indicates a relative percentage of error less than 6.5%. Keeping in mind that experiemntal results in general could experience an error of 5%. Therefore, the Brinkman model can be considered to be valid for nanoparticle volume fraction up 2%, which is the case of the present study.

**Figure 11:**
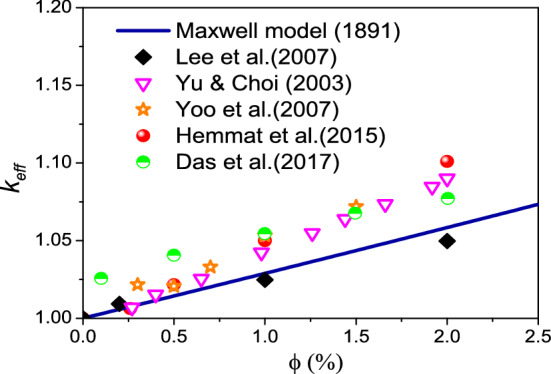
Comparison of the thermal conductivity by Maxwell^76^ at different naoparticle concentrations with previous experimental data.

In terms of thermal conductivity, Fig. 11 presents the data obtained for the effective thermal conductivity of Al_2_O_3_–water nanofluid containing various volume fractions of alumina particles along with the experimental results reported in the references^71–75^ and the results calculated based on the theoretical model by Maxwell^76^. It is evident from Fig. 11. that there exists good agreement between the reported experimental data and Maxwell model with a maximum percentage error of 4.7%, this indicates that Maxwell model can accurately predict the thermal conductivity of nanofluids with nanoparticle concentration up to 2.0 vol.%.

## Flow and temperature fields

In the following part, numerical simulations are carried out to examine the effect of Al_2_O_2_ nanoparticles and NEPCMs particles on the flow and temperature fields. Computations are made for different values of nanoparticle volume fraction of 0.43, 0.85, 1.27 and 1.69% and a wide range of Rayleigh numbers (10^4^ ≤ Ra ≤ 10^7^). Note that the nanoparticle volume fraction applies to both Al_2_O_3_ and NEPCM particles.

**Figure 12:**
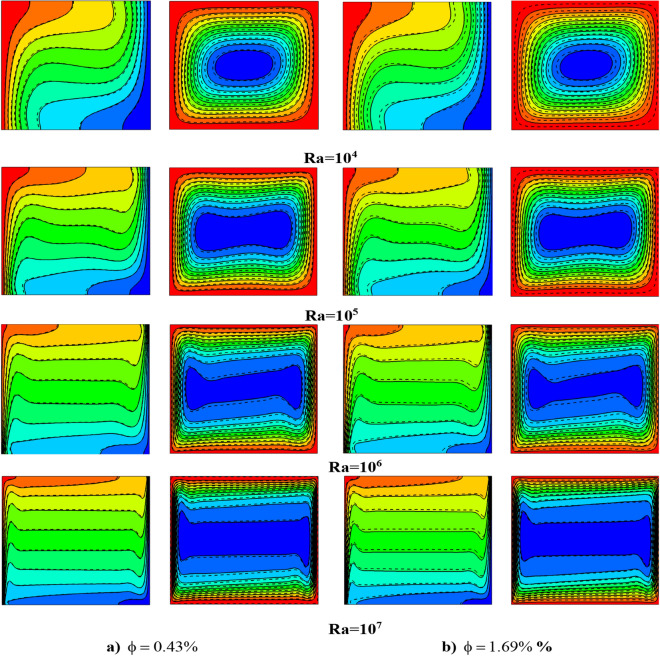
Streamlines and isotherms for LFTFs (solid lines) and Nanofluids (dashed lines) at different Rayleigh numbers.

Figure 12 depicts a comparison for isotherms and flow fields of LFTFs (solid lines) and nanofluids (dashed lines) for different values of the Rayleigh number in the range from 10^4^ to 10^7^, at the lowest and highest nanoparticle concentration in the considered range. For any given Rayleigh number, it is observed that the flow structure does not change regardless of the type of nanoparticles (with or without phase change) and nanoparticle volume fraction. However, the flow strength is affected by both nanoparticles type and concentration. It is observed that at low nanoparticle fraction, the percentage deviation between the maximum stream function values of LFTFs and nanofluids with respect to the nanofluid is 8.5%, 9.3%, 9.4%, and 8.9% for Ra = 10^4^, 10^5^, 10^6^, and 10^7^, respectively. While the percentage deviation at high nanoparticle fraction is 3.6%, 6.4%, 7.8%, and 5.5% for Ra = 10^4^, 10^5^, 10^6^, and 10^7^, respectively. This result indicates that the nanofluid flow is slower compared to the LFTFs flow, and hence, adding NEPCMs to the base fluid can provide better thermal performance. It should be noted that it was found that the base fluid flow is slightly slower compared to the nanofluid (streamlines and isotherms contours for the base fluid were not presented here). This result is expected since the increase in the thermal conductivity of the nanofluid is higher than the increase in the dynamic viscosity. In contrast, for the whole range of the Rayleigh numbers, the isotherms contours show slight deviation between the two fluids at φ = 0.43%. However, at φ = 1.69%, the deviation of LFTFs contours from that of nanofluids is noticeable in the cavity.

## Heat transfer

**Figure 13:**
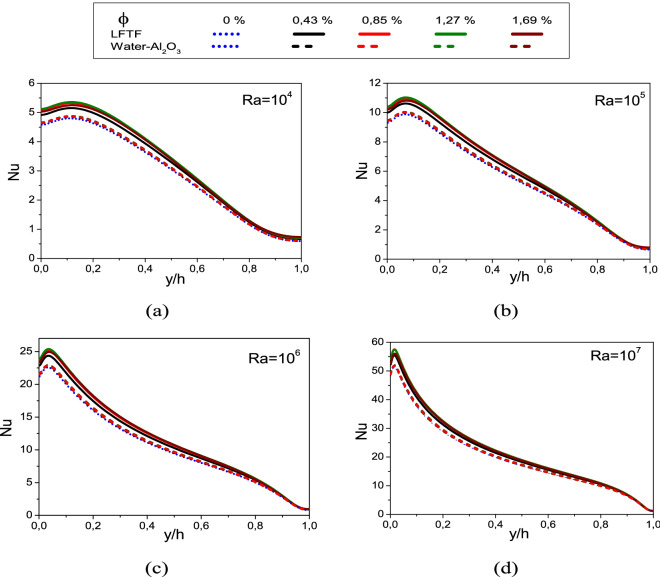
Variation of the local Nusselt number with nanoparticle volume fraction, (**a**) Ra = 10^4^, (**b**) Ra = 10^5^, (**c**) Ra = 10^6^, and (**d**) Ra = 10^7^.

**Figure 14:**
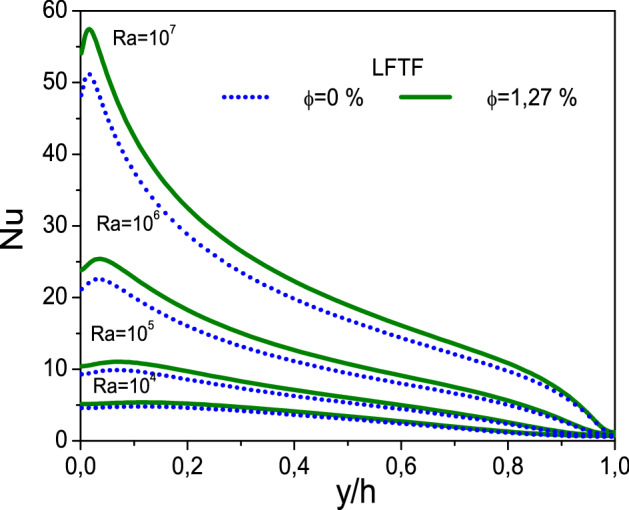
Variation of the local Nussel number of LFTFs at different Rayleigh numbers with $\upphi = 0\%$ and $\upphi = 1.27\%$.

Figure 13 illustrates the local Nusselt number along the hot wall as a function of Rayleigh number and nanoparticle volume fraction. Note that this figure is presented for the case of LFTFs and nanofluids. As seen, at higher Rayleigh number values, the convective heat transfer is strengthen in the cavity, and the flow velocity and the temperature gradient are therefore increased, As a result, the values of the Nusselt number are increased. The addition of Al_2_O_3_ nanoparticles to the base fluid has insignificant effect on the Nusselt number, and hence on the heat transfer. However, the addition of NEPCMs to the base fluid increases significantly the Nusselt number, which is found to exhibit a non-monotonic behavior on the nanoparticle volume fraction. It should be noted that the criterion for the heat transfer improvement in nanofluids is observed if the thermal conductivity is four times higher than dynamic viscosity. A further scrutiny of the curves in Fig. 14, however, divulges that the deviation between the Nusselt number of LFTFs form that of the base fluid increases as the Rayleigh number increases. For example, the maximum Nusselt number for LFTFs (Water) along the hot wall is 5.4 (4.8) and 57.5 (50.9) at Ra = 10^4^ and Ra = 10^7^, respectively.

**Figure 15:**
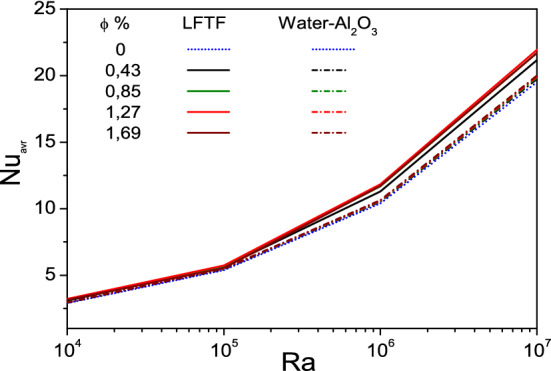
Variation of the mean Nusselt number with Rayleigh number as function of nanoparticle volume fraction.

Finally, the variation of the mean Nusselt number at the hot wall with nanoparticle volume fraction as a function of Rayleigh numbers is depicted in Fig. 15. The results are presented for the case of LFTFS and nanofluids. It is revealed that filling the cavity with nanofluids results in an insignificant increase in the mean Nusselt number; a maximum enhancement of 2.5% is observed at Ra = 10^7^ and φ = 1.69%. This clearly indicates that the mean Nusselt number is not sensitive to the addition of Al_2_O_3_ nanoparticles in the considered range. However, with increasing the Rayleigh number, the effect of adding NEPCMs to the base fluid on the mean Nusselt number tends to become more pronounced.; the Nusselt of LFTFs becomes significantly deviated from that of the base fluid (or nanofluid), particularly, at Ra = 10^7^ and $\upphi = 1.27\%$. It is worthy to note that the enhancement in the Nusselt number attains the lowest and highest values of 6.5% at Ra = 10^5^ and 13% at Ra = 10^6^.

To identify which physical property is behind such significant heat transfer enhancement in LFTFs over that for the nanofluids or the base fluid, the ratio of the mean Nusselt number of LFTFs to that of base fluid is calculated. In fact, for natural convection in a differentially heated square cavity, the correlation of the mean Nusselt number is given by^55^: ${\rm Nu}_{{{\rm av}}} ={\rm cRa}^{{\rm n}}$.

Therefore, the mean Nusselt number ratio between the LFTFS and the base fluid can be expressed as

25 $$\frac{{{\rm Nu}_{{{\rm avr,LFTFs}}} }}{{{\rm Nu}_{{{\rm avr,f}}} }}= \left( {\frac{{\upbeta _{{{\rm LFTFs}}} }}{{\upbeta _{{\rm f}} }}} \right)^{{\rm n}} \left( {\frac{{\uprho _{{{\rm LFTFs}}} }}{{\uprho _{{\rm f}} }}} \right)^{{{\rm 2n}}} \left( {\frac{{{\rm c}_{{{\rm p,LFTFs}}} }}{{{\rm c}_{{{\rm p,f}}} }}} \right)^{{\rm n}} \left( {\frac{{{\rm k}_{{{\rm LFTFs}}} }}{{{\rm k}_{{\rm f}} }}} \right)^{{{\rm 1 - n}}} \left( {\frac{{\upmu _{{{\rm LFTFs}}} }}{{\upmu _{{\rm f}} }}} \right)^{{{\rm - n}}}$$

Since the LFTFs is considered as an iso-density mixture, then the term $\left( {\frac{{\upbeta _{{{\rm LFTFs}}} }}{{\upbeta _{{\rm f}} }}} \right)^{{\rm n}} \left( {\frac{{\uprho _{{{\rm LFTFs}}} }}{{\uprho _{{\rm f}} }}} \right)^{{{\rm 2n}}}$ is considered equal to one. Thus, we have

26 $$\frac{{{\rm Nu}_{{{\rm avr,LFTFs}}} }}{{{\rm Nu}_{{{\rm avr,f}}} }}= \left( {\frac{{{\rm c}_{{{\rm p,LFTFs}}} }}{{{\rm c}_{{{\rm p,f}}} }}} \right)^{{\rm n}} \left( {\frac{{{\rm k}_{{{\rm LFTFs}}} }}{{{\rm k}_{{\rm f}} }}} \right)^{{{\rm 1 - n}}} \left( {\frac{{\upmu _{{{\rm LFTFs}}} }}{{\upmu _{{\rm f}} }}} \right)^{{{\rm - n}}} ={\rm C}_{{\rm n}}$$

From the above equation, it can be seen that the ratio is mainly affected by the relative increase in the specific heat, the relative enhancement in the dynamic viscosity, and the relative enhancement in the thermal conductivity. Note that for the nanofluids, this ratio is affected by the relative enhancement in the dynamic viscosity, the relative enhancement in the thermal conductivity, the relative decrease in the thermal expansion coefficient, the relative enhancement in the density, and the relative decrease in the specific heat.

It is clear that the LFTFS is beneficial for heat transfer only if C_n_ > 1. Adopting a value of ${\rm n = 0}{\rm .28}$ gives ${\rm 1}{\rm .01 \le }\left( {\frac{{{\rm c}_{{{\rm p,LFTFs}}} }}{{{\rm c}_{{{\rm p,f}}} }}} \right)^{{{\rm 0}{\rm .28}}} {\rm \le 1}{\rm .03}$. This indicates that the specific heat has negligible effect on the heat transfer enhancement in LFTFs. However, the ratio $({\text{c}}_{{\text{p,LFTFs}}} {\text{/c}}_{{\text{p,f}}} )_{{{\text{max}}}}^{0.28}$ is found almost equal to $({\text{Nu}}_{{\text{avg,nf}}} {\text{/Nu}}_{{\text{avg,f}}} )_{{{\text{max}}}}$. Therefore, this insignificant specific heat enhancement means that the viscosity increase is mainly surpassed by thermal conductivity increase in LFTFs.

## Conclusion

Throughout a careful examination on the comparison between LFTFs and nanofluids in improving the thermal efficiency of the conventional fluids, the following main conclusions are:

- For the case of LFTFs, it is found that the densities are not sensitive to the increase of nanoparticles volume fraction, and hence the mixture is considered as an iso-density. In contrast, the major issues in nanofluids is their stability (due to density variation), which remains challenging issue in nanofluids sedimentation.
- Unlike the specific heat capacities of nanofluids which decrease with increasing the nanoparticle volume fraction and are independent of temperature, the specific heat capacities of LFTFS increase significantly with nanoparticle volume fraction and temperature, particularly, during solid–liquid phase change.
- Dispersing NEPCMs in base fluid results in significant increase in both thermal conductivity and dynamic viscosity.
- Though the thermal conductivity is 15 times smaller than that of the base fluid, a significant enhancement in thermal conductivity is observed. This high enhancement in thermal conductivity of the LFTFs when compared to typical nanofluids is attributed to the high latent heat of fusion of the LFTFs.
- The LFTFs give substantial heat transfer enhancement compared to nanofluids. This enhancement is mainly due to the increase in the thermal conductivity.
